# Development of a Kinect Software Tool to Classify Movements during Active Video Gaming

**DOI:** 10.1371/journal.pone.0159356

**Published:** 2016-07-21

**Authors:** Michael Rosenberg, Ashleigh L. Thornton, Brendan S. Lay, Brodie Ward, David Nathan, Daniel Hunt, Rebecca Braham

**Affiliations:** School of Sport Science, Exercise and Health, University of Western Australia, M408 35 Stirling Highway, Crawley, WA, Australia 6009; Universite de Nantes, FRANCE

## Abstract

While it has been established that using full body motion to play active video games results in increased levels of energy expenditure, there is little information on the classification of human movement during active video game play in relationship to fundamental movement skills. The aim of this study was to validate software utilising Kinect sensor motion capture technology to recognise fundamental movement skills (FMS), during active video game play. Two human assessors rated jumping and side-stepping and these assessments were compared to the Kinect Action Recognition Tool (KART), to establish a level of agreement and determine the number of movements completed during five minutes of active video game play, for 43 children (m = 12 years 7 months ± 1 year 6 months). During five minutes of active video game play, inter-rater reliability, when examining the two human raters, was found to be higher for the jump (r = 0.94, p < .01) than the sidestep (r = 0.87, p < .01), although both were excellent. Excellent reliability was also found between human raters and the KART system for the jump (r = 0.84, p, .01) and moderate reliability for sidestep (r = 0.6983, p < .01) during game play, demonstrating that both humans and KART had higher agreement for jumps than sidesteps in the game play condition. The results of the study provide confidence that the Kinect sensor can be used to count the number of jumps and sidestep during five minutes of active video game play with a similar level of accuracy as human raters. However, in contrast to humans, the KART system required a fraction of the time to analyse and tabulate the results.

## Introduction

Active video games allow children the opportunity to expend more energy than they would when playing seated [1,2,3], and may potentially contribute to increases in physical activity and health [4]. Using full body motion to play active video games typically results in greater energy expenditure than just using upper limb movements [5]. However, there is little information on the classification of human movement during active video game (AVG) play in relationship to fundamental movement skills (FMS) [6], the basic building blocks of more specialised, complex skills used in organised and non-organised games, sports and leisure activities [7]. This may be due, in part, to the nature of the current generation of AVGs, as well as the time-consuming nature of traditional analysis of children’s movement in real time, or post hoc. Game technology has now progressed to a level where advanced motion monitoring techniques can be used to extract body segments from video and infrared cameras, with this data used to create an immersive game environment controlled by human motion [8,9,10]. However, the feasibility of using the game play technology to classify children’s movement rapidly whilst playing AVGs remains to be determined.

A number of factors influence the type of movement required of players within an AVG, including the type of game being played, whether the game is played competitively, cooperatively or solo, the motivation of players to engage in the movement while playing and the technology used to interact with the game [11, 12]. There is currently no requirement for the design of AVGs to include movements that resemble “real” actions to complete game play tasks and therefore, no incentive for their players to use traditional movement skills to achieve game outcomes. AVGs transform otherwise sedentary time playing video games into an opportunity to engage in physical activity [13], and given that one of the primary determinants of a physically active lifestyle is fundamental movement skill proficiency, it is important to understand how AVGs facilitate movement skill development for children. Currently, little is known about the movement skills performed during game play [6] and we do not have an efficient means of classifying these movements. A vital step towards understanding the impact AVGs have on skill development is, therefore, to identify the movement patterns and opportunities for skill performance during AVG play. No single method of identifying and assessing movement skills has been accepted as a ‘gold standard’, though many valid instruments for such tasks exist. While most instruments have a similar purpose, they differ in their constructs and assessment techniques [14, 15, 16]. Traditionally, identifying and assessing movement patterns have involved process-oriented, human rated assessment of the features, or processes within a particular skill performance, and are still the most commonly used movement skill assessment [15]. While these assessments are standardised in accordance with pre-determined criteria related to the characteristics of a skill, human variability makes it difficult to compare performances that are evaluated by different assessors.

Previous research has sought to use the principals of human driven movement assessment to understand the movement patterns of children during AVG play. Rosa and colleagues developed the Observational Tool for Active Gaming and Movement (OTAGM) to systematically assess movement patterns and characteristics of children during AVG play [17]. However, this tool relies on subjective, momentary time sampling techniques to generate data, and thus has the potential to over or under estimate a child’s actions. To reduce the subjectivity of movement classification, other studies have used valid methods of movement assessment, such as motion capture systems and force plates [18, 19]. Currently, these three-dimensional (3D) motion capture systems are the gold standard of accurately reporting movement kinematics, but are yet to be used to identify specific movement skills during AVG play, and are expensive, as well as time and labour intensive. Furthermore, these systems can constrain the movement of participants, limiting the opportunity to analyse movement in “free-play” settings; the advantage of using more observational tools. However, developments in action research through computer vision have advanced rapidly in recent years and in 2010, arrived at the consumer level with the release of the Microsoft Kinect Visual Optics Sensor [8, 20]. These technologies offer new approaches to measurement of movement kinematics during AVG play [21, 22].

### Sensor based human pose determination

Before the Microsoft Kinect™ Sensor was released in 2010, human pose was computed from complex analysis of captured video data [23]. For marker based optical motion capture systems, retro-reflective markers are attached to various body segments and landmarks (body joints), with proprietary software and some manual mapping of body segments needed in order to generate a visual human skeleton that can be used for learning and classification of human movements. The Microsoft Kinect Sensor was primarily designed for natural interaction in a computer game environment [24], and consists of an infrared laser emitter, an infrared camera and standard camera. Microsoft provides a software development kit (SDK) for the Kinect, allowing software engineers easily extract the 3D spatial and temporal data from 20 anatomical landmarks (joint centres). These 3D body joint positions can be described as a hierarchical tree structure with the root of the tree at the centre of the hips. They form the human pose needed for movement classification. Several researchers have found that better human poses can be determined with the use of two Kinect Sensors placed approximately at a 60-degree angle relative to a focal point (where the movement is performed). Such a configuration overcomes the occlusion problem encountered with one sensor while keeping the interference between the infrared signals to a minimum [10, 25].

Although the Kinect Sensor is unable to capture data at the same level or with the specificity of joint rotation as marker based optical motion capture systems, it has been shown to accurately match joint angles captured by them [26] and be used to identify kinematic and temporal parameters of skills, in both adult populations [26] and children [27]. The Kinect Sensor is therefore capable of being used to classify and potentially analyse movement, for the purpose of a movement proficiency assessment, and has successfully been implemented in the assessment of vertical jump development in children [27]. Indeed, this measurement tool is portable and, with small setup time, offers huge potential for objectively measuring the frequency and quality of human movement. Already, early applications of the Kinect Sensor technology include measuring falls risk (balance) in the elderly [28], passive in-home measurement of stride-to-stride gait [29] and even the analysis of children’s tantrum behaviour [30]. In addition to the above published research, there are many commercial applications developed for the Kinect, including remote E-Health monitoring [31] and the development of game-based balance rehabilitation tools [32].

The first step in applying this promising technology to the assessment of movement during AVG play is to determine whether specific FMS can be classified validly and reliably. In order to achieve this, we generated criteria to systematically classify jumps and side-steps, performed during AVG play. The aim of this study was, therefore, to validate the software’s ability to recognise and classify the FMS of jumping and side-stepping against human assessors, establish a level of agreement and count the number of movements completed during five minutes of AVG play.

## Methods

### Participants and settings

43 participants (11 females and 32 males) between the ages of 10 and 15 years were involved in this research. Descriptive information relating to participants can be found in Table 1. Participants were recruited through local advertisements and attended the University of Western Australia’s (UWA) AVG laboratory (A dedicated space designed to reflect a comparable child friendly home based gaming situation) in July 2013. Written informed consent from parents and children was obtained prior to the study, and this research was approved by the University of Western Australia Human Research Ethics Committee RA/4/1/4657. Participants were instructed to wear fitted clothing and removed footwear for the duration of the gaming sessions, in order to minimise the potential confounding effects this may have had on data collection [33].

**Table 1:** Descriptive information for participants.

	**N**	**Mean**	**Standard Deviation**
**Age (y/m)**	43	12 years 7 months	1 year 6 months
**Height (m)**	43	1.37	.13
**Weight (kgs)**	43	48.86	14.65

### Gaming session protocol

**Fig 1:**
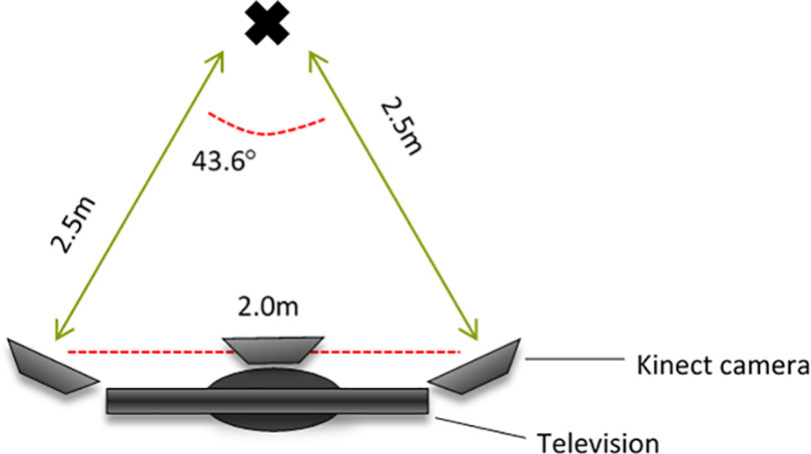
Laboratory set up of Kinect Sensors and Gaming Station.

Three children per session attended the AVG laboratory and were allocated to an individual AVG station. In line with previous research that has optimised Kinect sensor positioning to minimise occlusion, each AVG station was equipped with three Xbox360 Kinect Visual Optics Sensors in the layout shown in Fig 1 [10, 25]. The central Kinect controlled game play, while the two on either side of the station collected motion data. Participants were encouraged to remain in the data collection space for the duration of each task, with breaks offered in between, and time spent outside of the data collection space during each task was recorded, with this information being used to eliminate these periods of time from the data analysis. Data were collected under two conditions; a FMS condition and a game play condition. In the FMS condition, participants were asked to perform three jumps and twelve sidesteps (six to each side) in front of the visual optic sensors. In the game play condition, participants were encouraged to play a designated mini-game from within Kinect Adventures™; River Rush continuously, at their allocated AVG station for five minutes. Kinect Adventures is a full-body moving game requiring predominantly jumping and side-stepping [34] through 20 different adventures. Data for both conditions were recorded using software developed using the Kinect for Windows API, and discussed in more detail below.

## Motion data collection

**Fig 2:**
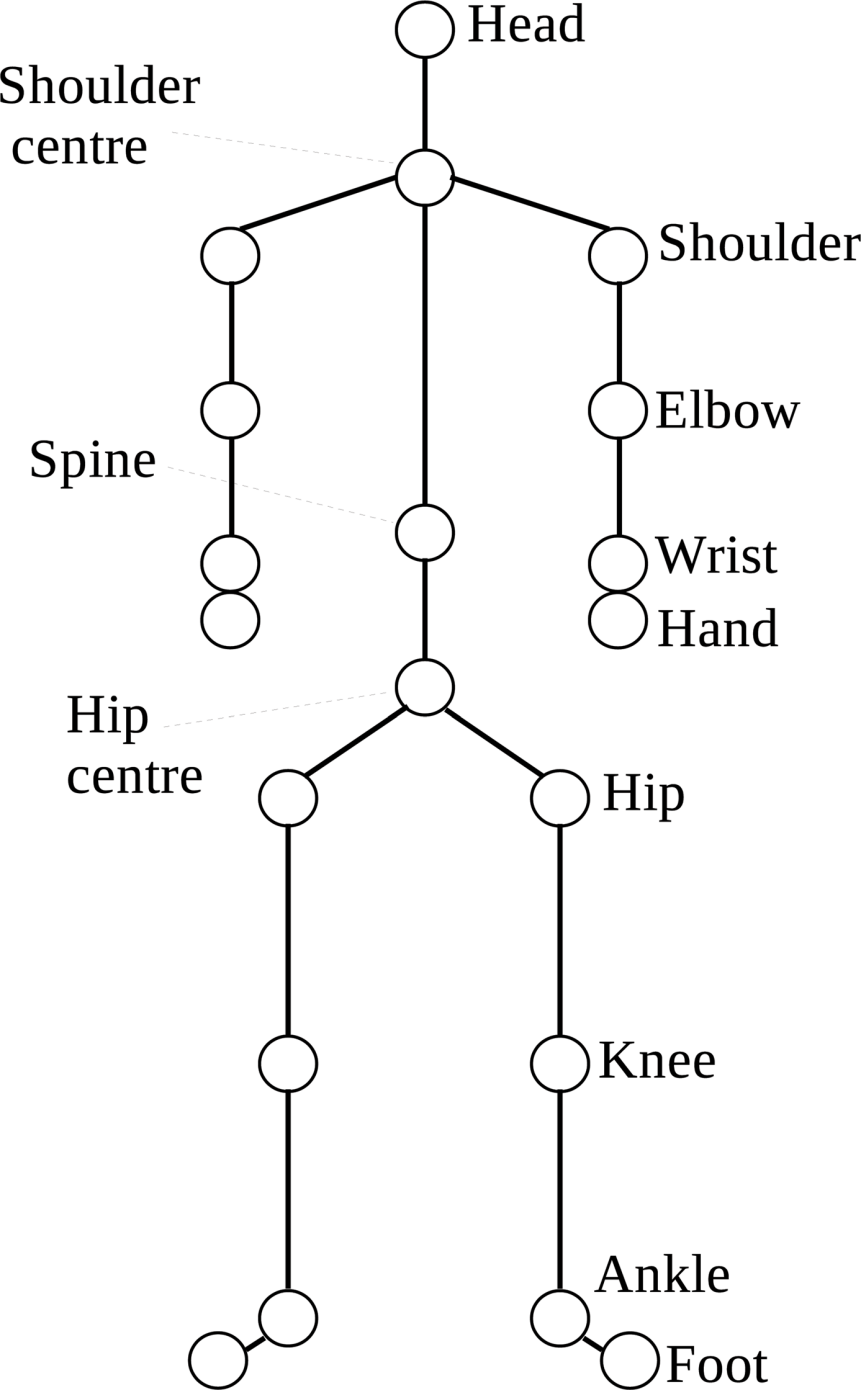
Tracked joint positions in the default position.

The structure of the motion data, and calibration of equipment for motion data collection, was based on previous research by Nathan and colleagues [35] existing Microsoft Kinect for Windows Software Development Kit (SDK) provides an Application Programming Interface (API) and access to the camera’s data streams, which produce 30 frames of data per second. The skeleton stream tracks twenty joints by default (Fig 2). Each joint position is represented as three floating point numbers that represent the XYZ coordinates in metres from each camera, with noise levels accounted for by a tracking state. A number of factors affect the noise level in the Kinect Skeleton Tracking Algorithm measurements: for example, extreme lighting, player size, player clothing and occlusion.

A tracking state that is represented by a validity character is provided for each Joint to indicate a level of confidence in the returned data. The state returned is either: Tracked, Inferred or Not Tracked. A Tracked state returns the highest confidence data since the Joint can be directly measured. An Inferred state indicates that the Joint data cannot be directly measured and instead is calculated from other Tracked Joints, while a Not Tracked state indicates that there is no data returned for the Joint.

A software system; the Kinect Action Recognition Tool (KART) constructed using the Kinect for Windows API was used to collect the motion data in each five minute period. The software system uses two Kinect cameras which are placed on either side of the player to minimise the effects of self-occlusion. The format of the motion data is a binary file that contains the twenty joint positions in XYZ coordinates, a validity character for each joint, twenty bone orientations in XYZ Euler angles and a timestamp for each frame. Combining the two camera’s data requires a calibration sequence in which the participant stands still with their arms straight and shoulders abducted to horizontal. This assumes that both cameras are at the same height. The distance between the left wrist and right wrist is measured and the difference in this value between the two cameras is required to be less than 10cm. The difference in position of the wrists in the X and Z coordinates is used to determine the angle that the second camera is facing compared to the first. Thus the joint positions from the second camera can be transformed into the coordinate system of the first. If the joint is being tracked by Camera One but not Camera Two then the value for Camera One is used and vice versa. If the joint position is valid in both cameras then the average of the two is taken and if it is invalid in both then Camera One's position is used. These different situations are represented in the validity character.

A graphical user interface (GUI) was developed within the KART, in order to facilitate the generation of simple rules by which FMS can be defined. The interface consists of several fields pertaining to aspects of the motion of a joint, which are combined with user imposed constraints on these fields to create conditions. For example, one could select ‘Joint Angle’ in conjunction with one of the 20 Kinect joints and then impose a condition on its value at the start of a particular FMS, such as constraining the value to be greater or less than some predetermined standard. Analogous conditions can be created to apply to other aspects of the joint data, and the rules can be linked by standard logical operators (‘AND’, ‘OR’, ‘IF-THEN’) intuitively from the GUI. The result of any such sequence of generated rule conditions is a description of the rule in plain English—useful for verifying the intent of the conditions—as well as a compact textual representation of the rule for later processing. The files corresponding to existing rules can be loaded into the GUI and modified, so they can be fine-tuned to match movements accurately.

Rules generated by the GUI can be applied to a skeleton stream recording in order to count the number of matches. This process is achieved by reading in the list of rules generated by the GUI, and then recursively applying the conditions in order of logical precedence (for example; ‘AND’ before ‘OR’) to a sliding window of frames from the movement. If the initial condition of a rule is matched by a given frame, a potential start frame is noted, and the rule is applied to subsequent frames until one is found that fails to meet the conditions in the rule (no match), or succeeds in meeting all conditions in the rule (valid match). The resulting sequence of recorded frames is saved as one match for the current rule, and the process is resumed at the last frame of the sequence. After the entire skeleton stream has been parsed against one rule, the process is repeated from the start for each subsequent rule. An output summary is generated after the process has completed, notifying the user of the number of matches with each rule and the time point at which they occur. This workflow is designed with flexibility in mind, and can be quickly repeated on different streams or with new rules.

### Data analyses

#### Human analysis

In line with Hallgren’s recommendations for establishing inter-rater reliability [36], a fully crossed design was implemented, in which two assessors, with experience in movement analysis, independently viewed skeletal motion data of each participant performing in both the FMS condition and game play condition, and recorded the occurrence of jumps and sidesteps across the two conditions. The two assessors then reviewed their recorded jumps and sidesteps to determine 100% agreement and the final number of jumps and sidesteps for each participant (as determined by 100% agreement of the raters) was then used to compare assessor ratings to the KART. This process involved both assessors viewing the movements that had been identified by one assessor, but not the other, and determining if they were included in the final count, alongside movements that had been identified by both assessors in the first instance.

#### Development of KART Rules

The development of Boolean rules, which were applied to data collected by the KART in the game play condition, required children to perform two specific FMS (jump and sidestep) in the FMS condition of data collection. This data was viewed, and commonalities in performance parameters between children were noted, with the minimum standard of performance used to inform the generation of rules for analysing all data, with some adjustment to minimise false positives and negatives. The final rules (see Table 2) were then applied to data collected during the game play condition, and a count of the number of times a jump and sidestep were performed were counted automatically by the software, for each participant.

**Table 2:** Final Boolean Rules as used in the game play condition.

**JUMP**
Vertical movement of the head changes at least 5cm in under 1 second
AND
Vertical movement of the left ankle changes at least 5 cm in under 1 second
AND
Vertical movement of the right ankle changes at least 5 cm in under 1 second
AND
Vertical movement of the hip centre changes at least 5cm in under 1 second
**SIDESTEP**
Horizontal Movement of the hip centre changes at least 10 cm in under .5 seconds
AND
Horizontal movement of the right foot changes at least 30 centimetres in under .3 seconds
AND
Horizontal movement of the shoulder centre changes at least 10 centimetres in under .5 seconds
OR
Horizontal Movement of the hip centre changes at least 10 cm in under .5 seconds
AND
Horizontal movement of the left foot changes at least 30 centimetres in under .3 seconds
AND
Horizontal movement of the shoulder centre changes at least 10 centimetres in under .5 seconds
AND
Horizontal movement of the right foot changes less than -25 centimetres in under .5 seconds

Correlations between assessors, as well as 100% agreement of assessors and the KART were compared using Intraclass Correlation Coefficients (ICC). For both conditions, a two-way random effects model with single measures accuracy was used. Percentage agreement was also calculated between assessors, as well as between 100% agreement of assessors and the KART across the FMS and game play conditions. There are no universally accepted criteria for reliability coefficients, but we have adopted the often used criteria recommended by Fleiss 1999 that considers ICCs of ≥0.75 excellent, <0.40 are poor, and those between the two ranges moderate [37].

## Results

### Inter-rater reliability

Inter-rater reliability for the two assessors was calculated within the game play condition for both the jump and sidesteps (Table 3). The ICC comparisons revealed reliability between assessors for both the jump (r = 0.94, p < .01) and sidestep (r = 0.87, p < .01) were excellent. Percentage agreement was higher for the jump (88.04%) than the sidestep (64.58%) between the two assessors.

**Table 3:** Average movement count, reliability analysis and percentage agreement for counting of the jump and sidestep between raters in the game play condition.

**Skill**	**Average Movement Count**		**Reliability Analysis**	**Percentage Agreement**
	Rater 1	Rater 2	Intraclass Correlation (95% CIs)	
**Jump**	20.19 ± 7.50	17.67 ± 7.67	0.94* (0.65–0.98)	88.04%
**SideStep**	6.55 ± 7.72	6.93 ± 8.00	0.92* (0.87–0.96)	64.58%

* Denotes significance at the p<0.01 level.

### 100% Rater agreement vs Kinect Action Recognition Tool in the fundamental movement skills condition

**Fig 3:**
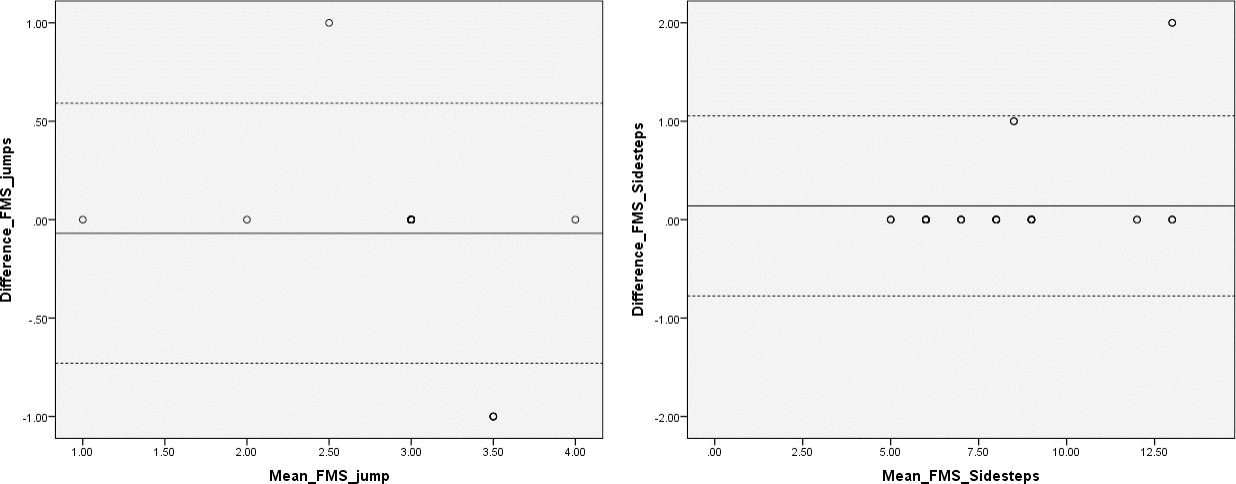
Bland-Altman plot for jump and sidestep: reliability of the Kinect Action Recognition Tool when compared to 100% human rater agreement in the fundamental movement skills condition. The dotted lines indicate limits of agreement (±1.96).

When the 100% rater agreement was compared to the KART count in the fundamental movement skill condition (Table 4), reliability was considered excellent between the assessor count and KART for the jump (r = 0.95, p < .01) and sidestep (r = 0.94, p < .01). Percentage agreement was also very high for both the jump (92.18%) and sidesteps (99.20%). Limits of agreement analyses were also calculated, the results of which are displayed in Fig 3.

**Table 4:** Average movement count, reliability analysis and percentage agreement for counting of the jump and sidestep between 100% rater agreement and KART in the fundamental movement skill condition (n = 43).

**Skill**	**Average Movement Count**		**Reliability Analysis**	**Percentage Agreement**
	Raters	System	Intraclass Correlation (95% CIs)	
**Jump**	2.95 ± 0.37	3.09 ± 0.68	0.83* (0.69–0.90)	92.18%
**SideStep**	8.23 ± 2.63	8.06 ± 2.38	0.94 *(0.92–0.96)	99.20%

* Denotes significance at the p<0.01 level.

### 100% Rater agreement vs Kinect Action Recognition Tool in the game play condition

**Fig 4:**
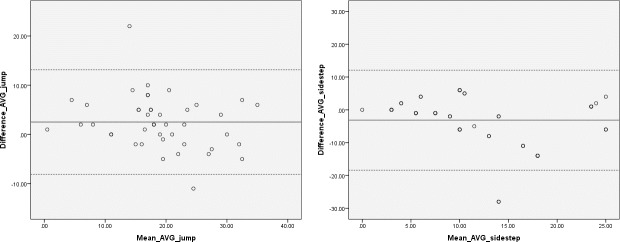
Bland-Altman plot for jump and sidestep: reliability of the Kinect Action Recognition Tool when compared to 100% human rater agreement in the game play condition. The dotted lines indicate limits of agreement (±1.96).

Lastly, 100% rater agreement was compared to the KART count in the game play condition (Table 5). Excellent reliability between assessors and the system was found for the jump (r = 0.84, p, .01) and moderate reliability for sidestep (r = 0.69, p < .01). Percentage agreement was higher for the jump (81.67%) than sidestep (58.63%) between the assessors and KART. Limits of agreement analyses were also calculated, the results of which are displayed in Fig 4.

**Table 5:** Total and average movement count, reliability analysis and percentage agreement for counting of the jump and sidestep between 100% rater agreement and KART in the game play condition (n = 43).

**Skill**	**Total Movement Count**		**Average Movement Count**		**Reliability Analysis**	**Percentage Agreement**
	Raters	System	Raters	System	Intraclass Correlation (95% CIs)	
**Jump**	873	945	20.30 ± 7.57	21.98 ± 10.02	0.84* (0.70–0.92)	81.67%
**SideStep**	189	243	11.11±7.80	14.29±8.98	0.69* (0.36–0.82)	58.63%

* Denotes significance at the p<0.01 level.

## Discussion

This study set out to establish the feasibility of the Kinect sensor to objectively and rapidly detect a child’s performance of a jump and sidestep, and to count the number of these movements within a five minute period of active video game play. The results suggest that the KART system was highly accurate in detecting isolated jumps and sidesteps in the FMS condition, and similar in accuracy to the two human raters during five minutes of game play. Both humans and KART had higher agreement for jumps than sidesteps in the game play condition. The results of the study provide confidence that the Kinect sensor can be used to count the number of jumps and sidesteps during five minutes of Kinect Adventures game play with a similar level of accuracy as human raters. Running the KART program, once the rules were established within it, was almost instantaneous, and the time taken to devise a skill count was maintained for each of the skills. This means the KART system took a fraction of the time to analyse movement when compared to the human analyses, which involved individually watching each skill one at a time to count. We are unaware of any other published research that has used the Kinect sensor to measure children’s movement during active video game play. For those interested in understanding the movement of children during active video game play, this approach has the potential to advance the way movement is recorded, analysed and reported, especially given the potential to expand the range of movements the KART can count across Kinect Adventures and other active video games.

While there were discrepancies between skills for movement detection accuracy, the higher accuracy of the KART to identify jumps compared with sidesteps in the game play situation was anticipated. The unique characteristics of a jump require systematic movement organisation to propel a person off the ground, using a two footed take off [21]. Sidesteps, however, are unilateral leg movements that can be undertaken by either side of the body [37]. As such, by nature they have less definite start and end points, and can be achieved with a wider variety of parameters, such as starting positions and width of the step. The challenge for the system was to detect sidesteps that weren’t part of maintaining the participant’s balance or incorporated into other motions; to achieve this, a more conservative sidestep criteria was used within the system. Nonetheless, the system counted more sidesteps than raters did (243 compared to 189), although the difference was relatively small compared with the overall number of movements (see S1 Data).

Our approach to extracting jumps and sidesteps relied upon developing a series of Boolean rules based upon how different body segments and joint angles changed within space and time. For example, a jump required both feet to leave the ground by a distance of at least five centimetres in under one second. This approach forces the software to extract features for both of the skills selected from the entire five minute data collection period based upon these pre-set values. Establishing the rules required, adjusting the parameters to reduce the number of false positives and negatives. While a satisfactory approach for the purpose of this study, it is possible that different results could be found for another group of children, or alternative game. However, we have no reason to believe the system would be less accurate with children playing alternative games, if jumping and sidestepping were performed with similar cadence and frequency. It is also possible that more sophisticated approaches based around machine learning could in the future provide better feature extraction across a greater range for movements, games and children.

While the accuracy of the human pose produced by the Kinect camera has been previously validated, this was typically using a stationary pose, or a slow controlled motion [21]. Children moving around playing active video games create additional problems for the Kinect sensor. The rate of capture for the Kinect sensor used in this study is 30 frames per seconds and this may impact the interpretation of more ballistic movements. The system developed as part of this study compensates in part for the frame rate, by using the two cameras to form skeletal positions. However, based on the recommendations of reliability established by Fleiss and colleagues [37], for the purpose of extracting and counting gross movements quickly and cheaply, the Kinect sensors are suitably accurate. The Kinect sensor also has a limited field of view, which individuals may leave and return, therefore impeding the KART’s ability to detect and analyse all of the potential movements occurring during game play. As such, care is required when establishing the parameters for data collection, to ensure individuals can participate fully in the tasks required for game participation, whilst remaining in the field of view.

The mini game River Rush within Kinect Adventures was selected, as previous research has shown the two movements most likely used in the game are jumping and sidestepping [34]. However, River Rush does not require a specific number of jumps or sidesteps to be completed to achieve the outcomes of the game, making it possible that the jumps and sidesteps counted by the human raters and the KART were unrelated to the game play. However, the purpose of this study was to determine if the KART could extract and count the number of movements from a stream of Kinect data, to a similar level of accuracy as human raters. Future research on matching the movement required/rewarded by the game and the actions of participants is warranted, in order to determine the extent to which AVGs engage children in the performance of movement skills. Literature indicates that the movements children perform during AVG play should be similar to those performed in real-life contexts, in order to promote the transfer of skill development in an AVG environment to physical activity behaviours [38]. However, there is limited research assessing how closely skills performed during AVG play reflect performance in physical activities outside AVG play, with the available evidence indicating that most skills performed in an AVG context are not reflective of proficient movement patterns in real life [17]. Contributing objective evidence to this body of research will now be somewhat easier, with the KART system’s ability to quickly identify, extract and count pre-set movements.

A limitation of the KART system in its current form is its inability to assess the quality of movement performed by children during active video game play. The criteria for recognising a jump requires minimal threshold body segment movements, but this only provides the minimum standard for a jump. It would be expected that developmental differences in the ability to perform jumps and sidesteps would be identified, as skill development is dependent on individual constraints such as age, as well as environmental and task constraints [39]. However, at this point, the system is limited by two movements and a narrow age group in the sample. Further evidence is required to ascertain whether the system is valid and reliable over different chronological ages and developmental stages, and able to qualitatively assess the movements being performed. The present study analysed children aged between 10 and 15 years, and from the perspective of the KART system and the use of Boolean rules to analyse this data, differences in body dimensions would play a large role in the performance and subsequent recognition of these movement skills, particularly in younger age groups. Another limitation of this research is the lack of information on whether jumps and sidesteps were required as part of the game. The KART was also designed to detect jumps and sidesteps from a series of generic rules. Greater accuracy may have occurred if individual variation was accounted for in this study; however, the KART would then require individual calibration to the unique parameters of FMS performance for each participant, which is both time and labour intensive. The overarching aim of the KART system is to minimise the labour required to identify movement.

This study has shown that the very technology used to engage children in active video game play can be used to count independently specific movements performed during game play. The results of this study also suggest that the KART system is similar in accuracy to human raters, although requires a fraction of the time and cost to complete the task. Analysing active video game play using the KART will provide valuable information to those interested in producing active video games with specific movements. As these types of systems evolve, it is anticipated that a focus on the quality of movement performed in front of the system will also eventuate, allowing a quick and efficient means of analysing the performance requirements, from a movement skill perspective, of active video games.

## Supporting Information

S1 Data KART and rater outputs. (XLSX)
